# Supplementary material for: Predicting visual function by interpreting a neuronal wiring diagram
Source: Nature. 2024 Oct 2;634(8032):113–23. doi: 10.1038/s41586-024-07953-5 (PMC11446822; doi:10.1038/s41586-024-07953-5)
Supplement: Supplementary file 6 — CRF and ERF predictions for individual TmY4 and TmY9 cells. Analogous to Supplementary Data 3, but for TmY target types. Shown are the top four monosynaptic pathways, the strongest pathway passing through each of the top ten intermediary types (ranking from Extended Data Fig. 7), and the trisynaptic pathway Tm1–TmY–Dm3–TmY (see the section entitled Prediction of spatial normalization). [file 41586_2024_7953_MOESM6_ESM.zip › DataS4/TmY9q/720575940624966328.pdf]

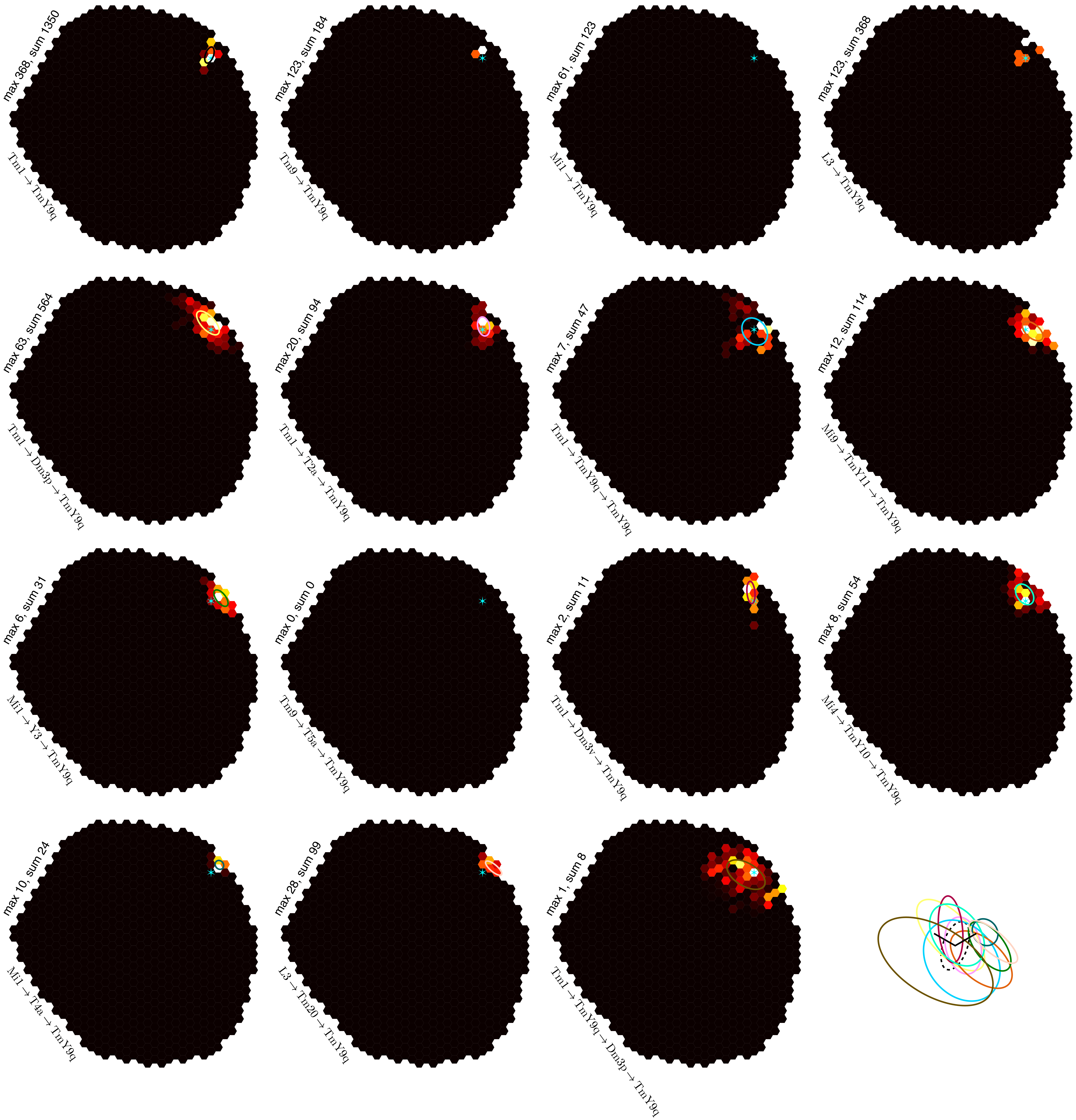

max 368, sum 1350
Tm1 → TmY9q
max 123, sum 184
Tm9 → TmY9q
max 61, sum 123
Mi1 → TmY9q
max 123, sum 368
L3 → TmY9q
max 63, sum 564
Tm1 → Dm3p → TmY9q
max 20, sum 94
Tm1 → T2a → TmY9q
max 7, sum 47
Tm1 → TmY9q → TmY9q
max 12, sum 114
Mi9 → TmY11 → TmY9q
max 6, sum 31
Mi1 → Y3 → TmY9q
max 0, sum 0
Tm9 → T5a → TmY9q
max 2, sum 11
Tm1 → Dm3v → TmY9q
max 8, sum 54
Mi4 → TmY10 → TmY9q
max 10, sum 24
Mi1 → T4a → TmY9q
max 28, sum 99
L3 → Tm20 → TmY9q
max 1, sum 8
Tm1 → TmY9q → Dm3p → TmY9q
